# POSTN⁺ cancer-associated fibroblast–CCL3⁺ macrophage crosstalk defines the immune-excluded tumor microenvironment in clear cell renal cell carcinoma

**DOI:** 10.1016/j.tranon.2026.102682

**Published:** 2026-01-24

**Authors:** Yingjian Wang, Bingtong Yue, Hongqiang Ni, Jinchun Chen, Run Shi, Zhe Wang, Xinglai Dai, Maolin Sheng

**Affiliations:** a Surgical Intensive Care Unit, Children's Hospital of Nanjing Medical University, Nanjing, China; b Department of Urology, The First Affiliated Hospital of Zhengzhou University, No. 1 Jianshe East Road, Zhengzhou, Henan 450052, China; c Department of Colorectal Surgery and Oncology, Key Laboratory of Cancer Prevention and Intervention, Ministry of Education, The Second Affiliated Hospital, Zhejiang University School of Medicine, Hangzhou, Zhejiang, 310009, China; d Department of Oncology, The First Affiliated Hospital of Nanjing Medical University, Nanjing, China; e Department of Oncology, The First Affiliated Hospital of Zhengzhou University, No. 1 Jianshe East Road, Zhengzhou, Henan 450052, China

**Keywords:** Clear cell renal carcinoma, Fibroblast, Macrophage, Periostin, Tumor microenvironment, Immunotherapy, Spatial transcriptomics

## Abstract

**Background:**

Clear cell renal cell carcinoma (ccRCC) frequently exhibits an immune-excluded tumor microenvironment (TME) that limits the efficacy of immune checkpoint blockade (ICB). However, the stromal–immune interactions responsible for this exclusion remain poorly understood.

**Methods:**

We integrated eight single-cell RNA sequencing datasets, two spatial transcriptomic datasets, and bulk transcriptomic cohorts to construct a comprehensive ccRCC TME atlas. Fibroblast subsets were characterized using clustering, trajectory, transcription-factor regulon, and gene-network analyses. Stromal–immune signaling was assessed using CellChat and NicheNet, and spatial colocalization patterns were validated by SpaGene analysis. Prognostic and therapeutic relevance were evaluated in TCGA-KIRC and ICB-treated cohorts.

**Results:**

Seven fibroblast subtypes were identified, among which periostin (POSTN)–positive cancer-associated fibroblasts (CAFs) were selectively enriched in tumors and exhibited strong activation of TGF-β, PI3K–AKT, and extracellular-matrix pathways. Trajectory and regulon inference revealed GATA6 as a key transcriptional regulator driving fibroblast differentiation toward this ECM-remodeling phenotype. Spatial analyses demonstrated that POSTN⁺ CAFs colocalized with CCL3-positive macrophages at the invasive front, forming a hypoxic, fibrotic niche that excluded CD8⁺ T cells. Ligand–receptor mapping identified reciprocal TGF-β, SPP1, and IL-6 signaling that reinforced fibro-myeloid activation. Activation of the POSTN⁺ CAF–CCL3⁺ macrophage axis correlated with poor survival and reduced response to ICB therapy.

**Conclusions:**

This study defines a spatially organized stromal–immune signaling axis that drives immune exclusion and immunotherapy resistance in ccRCC. Targeting the POSTN⁺ CAF–CCL3⁺ macrophage interaction offers a promising strategy to remodel the fibrotic barrier and restore antitumor immunity.

## Highlights

• POSTN⁺CAFs drive immunosuppression via CCL3⁺macrophages in ccRCC.
• Spatial mapping confirms POSTN⁺ CAF-CCL3⁺ macrophage colocalization.
• POSTN⁺ CAF-CCL3⁺ macrophage axis mediates T-cell exclusion in tumors.
• SpaGene analysis reveals ECM-immune programs in TLS⁺/TLS⁻ samples.
• Targeting POSTN⁺ CAFs may restore T-cell infiltration for immunotherapy.

## Graphical abstract

**Figure:**
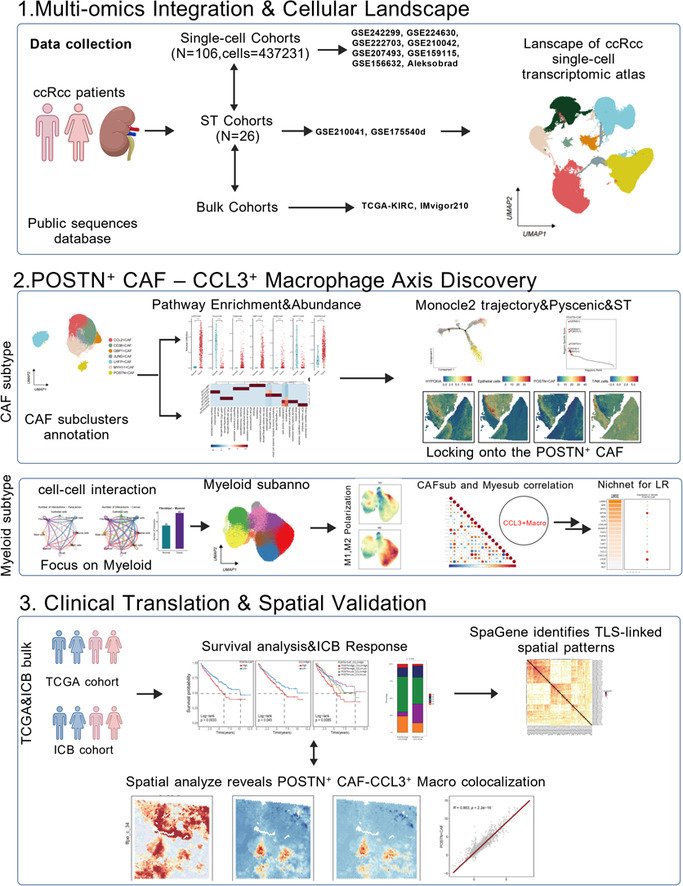

## Introduction

Kidney cancer represents roughly 4% of newly diagnosed cancers in the United States and ranks among the ten most common malignancies worldwide[1]. Almost 90% of kidney cancers are renal cell carcinoma (RCC), and about 70-80% of RCC cases are classified as clear cell renal cell carcinoma (ccRCC)[2]. Over the past decade, treatment for ccRCC has been revolutionized by the approval of immune checkpoint blockade (ICB) therapies, which have become first-line treatments for advanced ccRCC[[3], [4], [5]]. However, their clinical benefit remains limited by significant patient variability and both primary and acquired resistance[6]. A key mechanism of resistance is the immune-excluded phenotype, where physical and chemical barriers in the tumor microenvironment (TME) prevent CD8⁺ T cells from infiltrating the tumor core, hindering effective immune response[7,8]. Consequently, clarifying the mechanism that drive this barrier is crucial for overcoming ICB resistance in ccRCC.

Among stromal constituents, cancer-associated fibroblasts (CAFs) have emerged as the primary architects of this physical barrier[9]. Beyond producing extracellular matrix (ECM) components, CAFs actively remodel tissue architecture, secrete immunomodulatory ligands, and alter local metabolism to impede cytotoxic T-cell trafficking[[9], [10], [11]]. Recent single-cell and spatial transcriptomic studies have revealed striking CAF heterogeneity[12], identifying subsets such as myofibroblastic (myCAFs), inflammatory (iCAFs), and antigen-presenting (apCAFs) with distinct molecular signatures[13]. Specifically, myCAFs and iCAFs are typically enriched in immunosuppressive TMEs, driving ECM stiffening and secreting factors like IL-6 and CXCL12 that exclude T cells[13,14]. In contrast, apCAFs, which express MHC class II, CD74, and CIITA, play a role in antigen presentation and can activate T-cells to enhance antitumor immunity[13]. The balance between different CAF subsets significantly influences the immune landscape in cancer. Beyond these subsets, single-cell studies have uncovered novel marker-defined CAF subsets with unique immunoregulatory programs, many showing defined associations with ICB responsiveness[[15], [16], [17]].

Furthermore, CAFs rarely act alone. The physical barriers they form require the close cooperation and maintenance of innate immune cells, particularly macrophages[18]. Emerging evidence indicates that CAFs and macrophages engage in reciprocal molecular communication that reinforces an immunosuppressive microenvironment[19,20]. For example, CAFs secrete chemokines such as CCL2, CXCL12, and IL-6 to recruit and polarize macrophages toward an M2-like immunosuppressive phenotype[21,22]. In turn, M2-like Macrophages release growth factors and matrix-remodeling molecules, including TGF-β, PDGFB, and SPP1, which reinforce CAF activation and ECM deposition[23]. This reciprocal signaling establishes a CAF-macrophages axis that functions as a self-perpetuating feedback circuit, amplifying stromal fibrosis, suppressing T-cell infiltration, and promoting immune evasion[18]. More importantly, this crosstalk represents a therapeutically actionable target within the tumor microenvironment. For example, Long et al. reported that DAB2⁺ macrophages and FAP⁺ CAFs synergistically form a dense tumor barrier in HCC[24]. This finding indicates a new therapeutic paradigm where targeting CAFs or macrophages alone may be insufficient and disrupting their cooperative axis is the key to restoring immune infiltration.

Although recognized as a key stromal-immune circuit, the CAF-macrophages axis remains poorly defined in ccRCC, particularly regarding its spatial organization and function. Existing bulk and single-cell studies lack integrated multi-omics and spatial analyses to resolve stromal-immune communication in the tumor microenvironment. This study systematically addresses this critical knowledge gap. By integrating multi-cohort scRNA-seq, spatial transcriptomics, and in-depth computational biology analyses (WGCNA, pySCENIC, Monocle2), we identified a late-stage CAF subpopulation in ccRCC characterized by POSTN expression. Crucially, we discovered for the first time that this POSTN⁺ CAF subpopulation spatially co-localizes with a specific CCL3⁺ macrophage subpopulation at the tumor-infiltrating margin. This POSTN⁺ CAF-CCL3⁺ macrophages axis collaboratively constructs a dense ECM barrier, leading to CD8⁺ T-cell exclusion. Finally, in an ICB-treated cohort, the activation of this axis was confirmed as a core driver of immunotherapy resistance and poor prognosis. Our study provides new insights into the immune exclusion mechanism in the ccRCC TME and establishes a theoretical foundation for developing combination therapeutic strategies targeting this axis.

## Material and methods

### Data collection

The scRNA-seq datasets (GSE242299, GSE224630, GSE222703, GSE210042, GSE207493, GSE159115, GSE156632, and Aleksobrad) were obtained from the GEO database and GitHub (https://github.com/Aleksobrad/single-cell-rcc-pipeline). Bulk transcriptome data (TCGA-KIRC) were obtained from the UCSC Xena database. Spatial transcriptomics data (GSE210041 and GSE175540) were obtained from the GEO database.

### Single-cell data processing and differential expression analysis

The Seurat (v4.3.0) R package was applied for downstream analyses. Low-quality cells (UMIs <500 or >15% mitochondrial genes) were filtered out, and potential doublets were identified using DoubletFinder (v2.0.4). Batch effects among samples were corrected using the Harmony integration algorithm. The top 2000 variable genes were selected with *FindVariableFeatures* for dimensionality reduction by principal component analysis (PCA). Based on the top 50 PCs, cell clustering was performed using *FindNeighbors* and *FindClusters*, and results were visualized with UMAP. Major cell lineages were annotated by canonical markers, including Epithelial cells(EPCAM, KRT18, KRT8, CRYAB), B/Plasma cells (CD79A, CD79B, MS4A1, IGKC), Endothelial cells (PECAM1, VWF, FLT1, PLVAP), Prolif (MKI67, TOP2A, CENPF), Fibroblasts (COL1A1, COL1A2, DCN, ACTA2), Myeloid cells (CD68, C1QB, LYZ, LAMP3), Mast cells (CPA3, TPSAB1, KIT, MS4A2), and T/NK cells (CD3D, CD3E, CD3G, NKG7). Differentially expressed genes (DEGs) were identified with *FindAllMarkers* using thresholds of *min.pct = 0.25, logfc.threshold = 0.25*, and *only.pos = TRUE*. Statistical significance was evaluated with the Wilcoxon rank-sum test adjusted by the Benjamini–Hochberg method.

### Gene set activity scoring

Single-cell gene signature scoring was performed using the AUCell(v1.20.2) package, and gene sets were ranked with the AUCell_buildRankings function. Signature enrichment differences were tested via a two-tailed Wilcoxon rank-sum test with false discovery rate (FDR) correction. All gene sets used are listed in *Supplementary Table 1*.

### Pathway and cytokine signaling analysis

CAF-related pathway activities were inferred using PROGENy. Scores were computed with run_aucell from the progeny (v1.24.0) package, based on the PROGENy reference network. The top 1000 target genes were used following standard single-cell recommendations.

### Functional enrichment analysis

Pathway enrichment and gene set enrichment analysis (GSEA) were conducted with the clusterProfiler (v4.6.2) package using KEGG reference pathways. Terms with an adjusted *p* value <0.05 were considered statistically significant.

### Trajectory inference

CAF differentiation trajectories were reconstructed using Monocle2 (v2.26.0). Prior to trajectory analysis, the fibroblast subset was batch-corrected using Harmony, and cell clusters were identified based on the Harmony reduction with FindNeighbors (dims = 1:20) and FindClusters (resolution = 0.3). The top 100 differentially expressed genes distinguishing CAF subclusters were used to define the trajectory. Dimensionality reduction was performed using DDRTree (max_components = 2), pseudotime-associated genes were identified by differentialGeneTest, and branch-dependent programs were analyzed using BEAM and visualized with plot_genes_branched_pseudotime.

### Transcription factor regulon inference

Transcriptional regulatory networks were reconstructed using pySCENIC, which calculates regulon activity based on co-expression and motif enrichment. Subtype-specific transcription factors were identified by the Wilcoxon test, and regulon specificity scores were computed using Jensen–Shannon divergence via the philentropy (v0.6.0) package.

### Cell–cell communication analysis

Potential interactions between cell types were predicted using single-cell RNA sequencing data and the CellChat software package (v2.2.0). CellChatDB.human is the reference database for ligand-receptor interactions. We used a significance threshold with a P-value cut-off of 0.05 in this database to predict cell–cell interactions between different cell types. To further characterize crosstalk between POSTN⁺ CAFs and CCL3⁺ macrophages, we employed the NicheNet package to infer ligand–target interactions. The top 20 ligands and 100 downstream targets were used to compute ligand–target activity (score range 0–1). Heatmaps displaying average expression across subtypes were generated after scaling.

### Bulk RNA-seq deconvolution

Cell-type composition in bulk RNA-seq and microarray datasets was estimated using BayesPrism. Custom signature matrices were derived from the single-cell reference. Quartile normalization was disabled for RNA-seq but enabled for microarray data. Estimated cell fractions were correlated using Spearman’s test, and visualized with the corrplot (v1.6.10) package.

### Survival and clinical correlation analysis

Overall survival was analyzed using the survival R package. Hazard ratios (HRs) and 95% confidence intervals (CIs) were calculated via Cox proportional hazards regression. Kaplan–Meier curves were plotted with survfit, and group comparisons were made using the log-rank test. Response rates to immune checkpoint blockade (ICB) were compared by the χ² test.

### Spatial transcriptomics analysis

Spatial gene–spot matrices from ST and Visium data were analyzed using Seurat (v4.3.0). Spots expressing fewer than 200 genes or genes detected in fewer than three spots were filtered out, and data were normalized using LogVMR. PCA was performed using the first 30 principal components, followed by spatial resolution enhancement with BayesSpace (v1.19.4) using the spatialEnhance and enhanceFeatures functions. Single-cell–derived signature scores were mapped to ST data using AddModuleScore and visualized with SpatialFeaturePlot.

Spatial gene expression patterns were identified using the SpaGene algorithm, which consistently detected three spatial modules per sample. For each module, the top 100 genes with the highest spatial weights were extracted. Cross-section reproducibility was assessed by pairwise Jaccard similarity based on gene overlap, with similarity matrices visualized using ComplexHeatmap (v2.14.0). A Jaccard similarity threshold of ≥0.4 was empirically selected to define conserved spatial modules, and the three patterns were reproducibly identified across all 24 tissue sections.

### Statistical analysis

All statistical analyses were performed in R (v4.3.1). Correlations were assessed using Spearman’s test. Group comparisons employed Kruskal–Wallis and Wilcoxon rank-sum tests with Benjamini–Hochberg correction for multiple testing. A p value < 0.05 was considered statistically significant.

## Results

### Single-cell landscape of clear cell renal cell carcinoma delineates major stromal and immune compartments

**Fig. 1:**
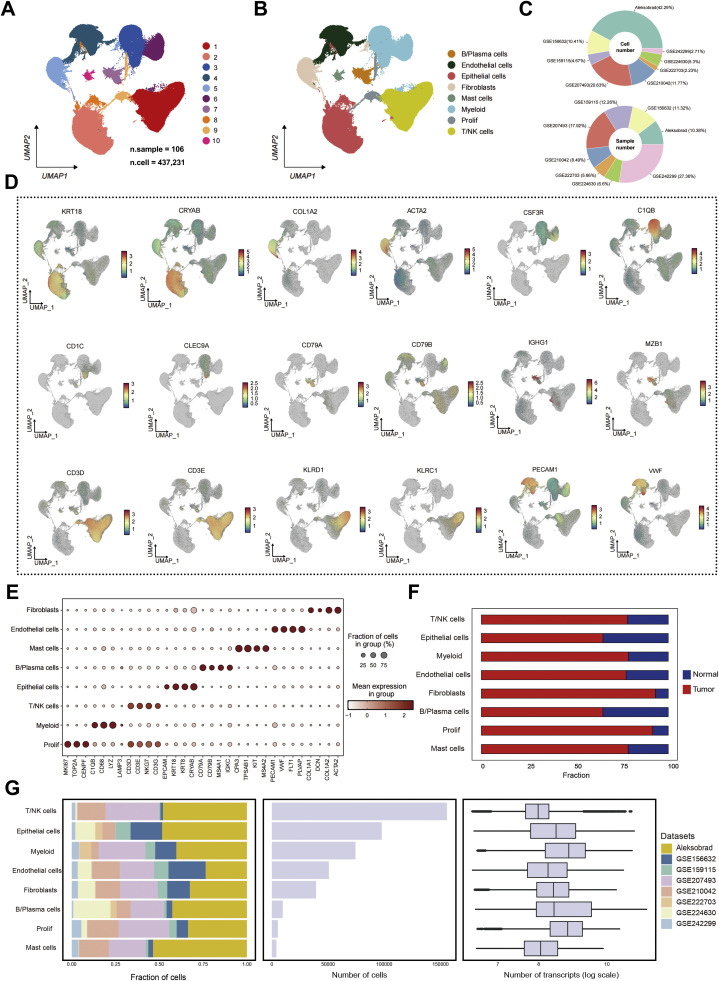
Single-cell landscape of ccRCC delineates major stromal and immune cell compartments. **(A)** Uniform manifold approximation and projection (UMAP) plot of integrated single-cell RNA-seq profiles from 106 samples (total 437,231 cells) after batch correction, showing 10 major cell clusters. **(B)** Annotation of major cell types, including B/plasma cells, endothelial cells, epithelial cells, fibroblasts, mast cells, myeloid cells, proliferating cells, and T/NK cells, based on canonical marker expression. **(C)** Distribution of cells and samples among integrated GEO datasets, indicating dataset composition across GSE149614, GSE151530, GSE156625, GSE189903, GSE202642 and Aleksobrad. **(D)** Expression patterns of representative marker genes used for cell-type identification: epithelial (KRT18, CRYAB), fibroblast (COL1A2, ACTA2), myeloid (CSF3R, C1QB), B/plasma cells (CD79A, CD79B, IGHG1, MZB1), T/NK cells (CD3D, CD3E, KLRD1, KLRC1), and endothelial (PECAM1, VWF). **(E)** Dot plot summarizing canonical marker expression across the 8 major cell lineages, showing both the percentage of cells expressing each marker (dot size) and average expression level (color scale). **(F)** Comparison of cellular composition between tumor and adjacent normal tissues, revealing increased proportions of fibroblasts, myeloid cells, and T/NK cells in tumor samples. **(G)** Summary of cellular proportions (left), total cell counts (middle), and sequencing depth (right) across datasets, confirming balanced data integration and consistent transcript coverage.

To comprehensively characterize the tumor microenvironment (TME) of clear cell renal cell carcinoma (ccRCC), we integrated six publicly available single-cell RNA sequencing (scRNA-seq) datasets encompassing 106 samples and a total of 437,231 cells after quality control (Fig. 1A). Batch effects across datasets were effectively corrected using the Harmony algorithm, resulting in a unified cellular atlas visualized by uniform manifold approximation and projection (UMAP). The integrated data revealed ten major transcriptional clusters (Fig. 1A), which were subsequently annotated into seven principal cell lineages, including T/NK cells, myeloid cells, B/plasma cells, endothelial cells, fibroblasts, epithelial/tumor cells, proliferating cells, and mast cells (Fig. 1B).

The integrated dataset covered multiple independent cohorts (Fig. 1C), ensuring robust representation of both tumor and adjacent normal tissues. Cell-type identities were assigned based on canonical lineage markers (Fig. 1D–E), such as CD3D and CD3E for T cells, CSF1R and C1QB for myeloid cells, COL1A2 and ACTA2 for fibroblasts, PECAM1 and VWF for endothelial cells, and CA9 and KRT18 for epithelial tumor cells. This annotation framework accurately recapitulated known components of the ccRCC microenvironment.

Comparative analysis of cell-type proportions between tumor and normal samples revealed marked compositional differences (Fig. 1F). Tumor tissues displayed a significant expansion of fibroblasts and myeloid cells, accompanied by a reduction in T/NK cells and endothelial cells, highlighting a fibro-inflammatory remodeling pattern characteristic of immune-excluded phenotypes. Quality control metrics confirmed consistent sequencing depth and cell-type distribution across datasets (Fig. 1G). Collectively, these data establish a high-resolution single-cell atlas of ccRCC that captures both immune and stromal heterogeneity, providing a foundation for dissecting cell–cell interactions within the tumor microenvironment.

### Identification and molecular profiling of POSTN⁺ CAFs as ECM-remodeling fibroblasts

**Fig. 2:**
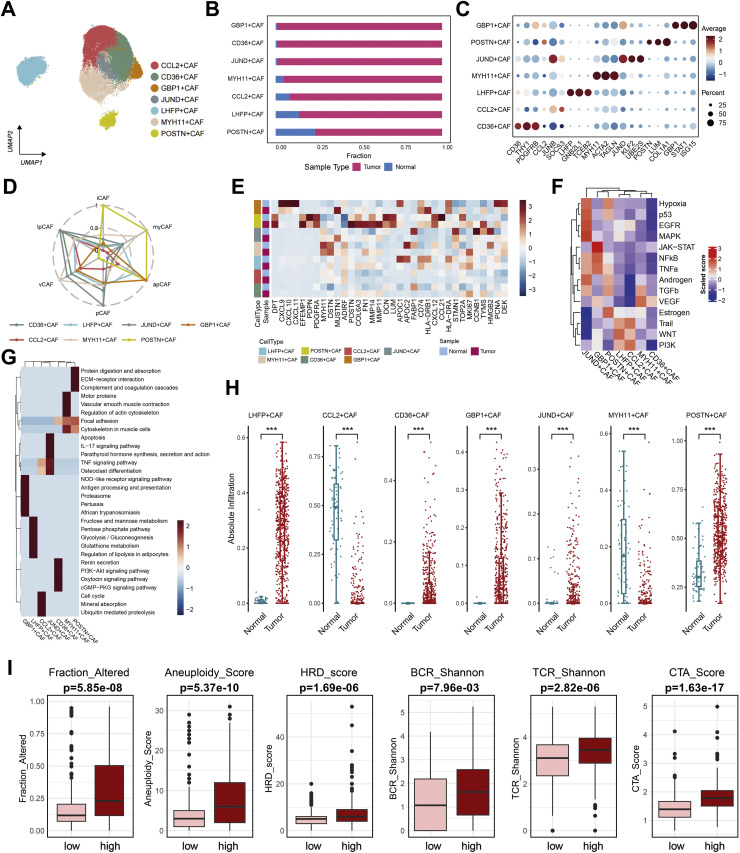
**Characterization of distinct CAF subtypes and their molecular features in ccRCC. (A)** UMAP visualization showing seven transcriptionally distinct CAF subsets (CCL2⁺, CD36⁺, GBP1⁺, JUND⁺, LHFP⁺, MYH11⁺, and POSTN⁺ CAFs) identified from integrated single-cell data. **(B)** Proportions of each CAF subset in tumor versus adjacent normal tissues, revealing marked expansion of POSTN⁺ CAFs within tumor samples. **(C)** Dot plot of representative marker genes for each CAF subset, displaying both the percentage of expressing cells (dot size) and mean expression level (color scale). **(D)** Radar plot summarizing functional signatures of the seven CAF subtypes, including inflammatory (iCAF), vascular (vCAF), proliferative (pCAF), myofibroblastic (myCAF), and matrix-associated (mCAF) features. **(E)** Heatmap showing pathway activity scores across CAF subsets, highlighting enhanced extracellular matrix (ECM) organization, collagen synthesis, and contractile programs in POSTN⁺ CAFs. **(F)** Signaling pathway activity comparison across CAF subsets using PROGENy, demonstrating prominent activation of TGF-β, PI3K/AKT, and hypoxia pathways in POSTN⁺ CAFs. **(G)** KEGG pathway enrichment analysis of POSTN⁺ CAF–specific genes, indicating enrichment in ECM–receptor interaction, focal adhesion, TGF-β signaling, and oxidative stress–related pathways. **(H)** Deconvolution analysis of bulk RNA-seq data showing significantly higher infiltration scores of five CAF subsets in tumor tissues compared with normal tissues (Wilcoxon test, ***p < 0.001). **(I)** Association between POSTN⁺ CAF enrichment and genomic instability metrics in the TCGA-KIRC cohort. Tumors with high POSTN⁺ CAF abundance exhibit increased fraction of genome altered, aneuploidy, homologous recombination deficiency (HRD), and elevated BCR/TCR diversity as well as cancer-testis antigen (CTA) activity, suggesting a link between POSTN⁺ CAF expansion and genomic dysregulation.

To delineate the phenotypic heterogeneity of fibroblasts within the ccRCC microenvironment, we extracted all fibroblast clusters from the integrated single-cell dataset for subclustering analysis. Unsupervised clustering identified seven transcriptionally distinct CAF subsets, defined by their dominant marker genes: CCL2⁺ CAFs, CD36⁺ CAFs, GBP1⁺ CAFs, JUND⁺ CAFs, LHFP⁺ CAFs, MYH11⁺ CAFs, and POSTN⁺ CAFs (Fig. 2A–C). Among these, POSTN⁺ CAFs were markedly enriched in tumor samples, whereas contractile MYH11⁺ CAFs and quiescent LHFP⁺ CAFs predominated in adjacent normal tissues (Fig. 2B). Marker-based annotation and hierarchical comparison revealed that POSTN⁺ CAFs expressed high levels of COL1A2, ACTA2, and TAGLN, reflecting a myofibroblast-like phenotype with strong extracellular matrix (ECM) remodeling capacity (Fig. 2C).

To functionally classify these subtypes, we compared CAFs against established fibroblast phenotypes including iCAF, myCAF, apCAF, and vCAF programs. Radar plot analysis showed that POSTN⁺ and CD36⁺ CAFs were enriched for myCAF-associated signatures, whereas CCL2⁺ and GBP1⁺ CAFs exhibited iCAF-like inflammatory features, characterized by cytokine secretion and chemokine activity (Fig. 2D). Pathway scoring further demonstrated that POSTN⁺ CAFs displayed the strongest activation of ECM–receptor interaction, focal adhesion, PI3K-AKT, and TGF-β signaling pathways (Fig. 2E–G), indicating a highly pro-fibrotic and matrix-restructuring phenotype. Notably, POSTN⁺ CAFs also showed elevated activity in hypoxia, STAT3, and p53 signaling modules (Fig. 2F), suggesting that hypoxic stress and oncogenic signaling may drive their differentiation within the tumor stroma.

Comparative quantification revealed that all seven CAF subsets were significantly expanded in tumor tissues relative to adjacent normal kidney (Fig. 2H), with POSTN⁺ CAFs showing the highest infiltration fraction (*p* < 0.001). To explore the biological consequences of CAF activation, ccRCC samples were stratified according to CAF signature enrichment. Tumors with high POSTN⁺ CAF scores exhibited elevated aneuploidy, homologous recombination deficiency (HRD), and genomic alteration burdens, along with increased BCR/TCR diversity and cancer-testis antigen (CTA) expression (Fig. 2I). This association may reflect a permissive relationship between fibrotic stromal remodeling and genomic instability, potentially mediated by hypoxic and stress-enriched microenvironmental conditions, although causality cannot be inferred from the current data. These findings suggest that POSTN⁺ CAF expansion, is associated with both genomic instability and immune diversification, collectively shaping a fibrotic and immunosuppressive tumor niche.

Collectively, these results identify POSTN⁺ CAFs as the dominant ECM-remodeling fibroblast population in ccRCC, characterized by strong activation of TGF-β/PI3K-AKT/STAT3 pathways and preferential accumulation within tumor regions. This CAF subtype likely serves as a structural scaffold and paracrine regulator that orchestrates stromal fibrosis and immune exclusion in the ccRCC microenvironment.

### Network-based analysis identifies POSTN⁺ CAF-associated gene modules in ccRCC fibroblasts

**Fig. 3:**
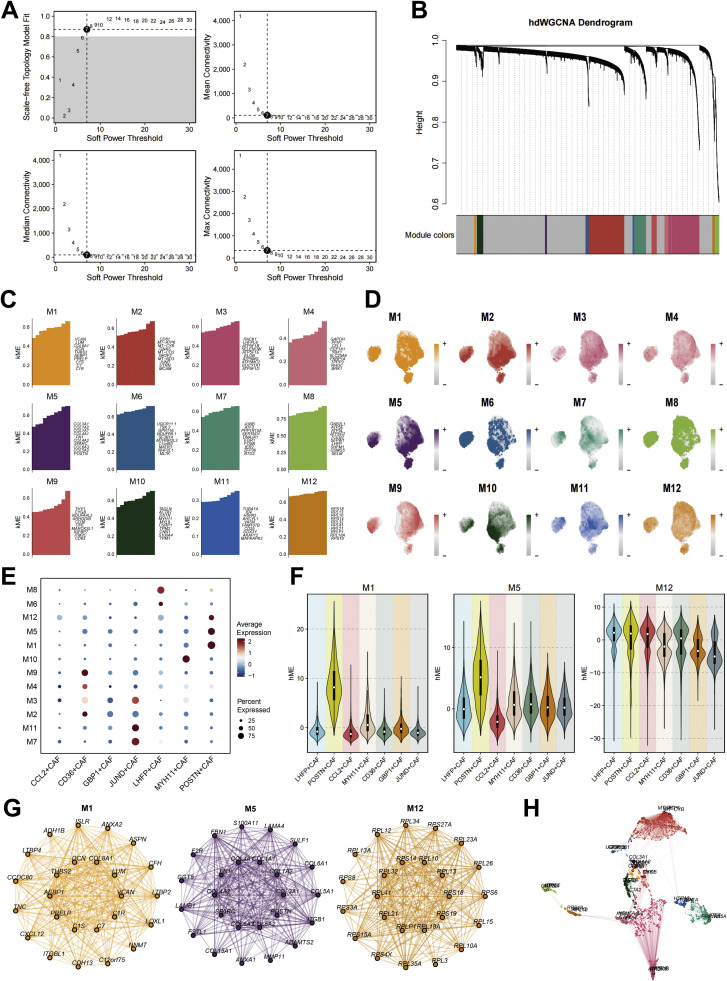
Weighted gene co-expression network analysis (WGCNA) reveals transcriptional modules associated with distinct CAF subsets. (**A)** Selection of soft-thresholding power for network construction based on scale-free topology model fit and connectivity analysis. Power value = 9 was chosen as optimal. **(B)** Hierarchical clustering dendrogram of gene co-expression profiles generated by WGCNA, showing 12 distinct gene modules (M1–M12) represented by unique colors. **(C)** Module eigengene (ME) values for each CAF subset, summarizing expression correlations between individual modules and specific CAF phenotypes. **(D)** UMAP visualization displaying spatial distribution and activity of each co-expression module (M1–M12) across all CAF subsets. **(E)** Dot plot illustrating average expression and proportion of module-enriched genes in each CAF subset, highlighting preferential enrichment of modules M1, M5, and M12 in POSTN⁺ CAFs. **(F)** Violin plots showing module eigengene values (MEs) of the top three CAF-enriched modules (M1, M5, and M12) across the seven CAF subsets. POSTN⁺ CAFs exhibit the strongest activation of these key transcriptional programs. **(G)** Co-expression networks of hub genes within the M1, M5, and M12 modules, identifying hub genes such as COL1A1, FBLN1, and IGFBP7 involved in extracellular matrix (ECM) remodeling and fibroblast activation. **(H)** Visualization of hub gene co-expression networks of all 12 modules, confirming their co-expression patterns within POSTN⁺ CAF populations.

To delineate the transcriptional programs underlying CAF heterogeneity in ccRCC, we performed weighted gene co-expression network analysis (WGCNA) on fibroblast transcriptomes (Fig. 3A). A soft threshold power of 7 was selected to achieve scale-free topology (R²= 0.9), identifying 12 distinct co-expression modules (M1-M12) with unique gene expression profiles (Fig. 3B). Module significance analysis revealed characteristic gene expression and connectivity patterns across these modules, with certain modules showing particularly strong intramodular connectivity (Fig. 3C). UMAP projection further demonstrated the spatial distribution of genes within each module, revealing distinct clustering patterns that reflect the functional coherence of co-expressed genes (Fig. 3D).

Correlation analysis between CAF subtypes and module eigengenes identified M1, M5, and M12 as the modules most strongly associated with POSTN⁺ CAFs, showing significantly higher expression levels in this specific CAF subpopulation compared to other fibroblast subtypes (Fig. 3E-F). Hub gene network visualization highlighted key regulators within these POSTN⁺ CAF-associated modules, revealing intricate co-expression relationships among the top connected genes (Fig. 3G). Notably, POSTN, COL1A1, COL3A1, FN1, and SPARC emerged as central nodes with high connectivity scores, suggesting their pivotal roles in maintaining the network structure. The comprehensive co-expression network encompassing all 12 modules demonstrated the overall architecture of transcriptional coordination in ccRCC fibroblasts, with the POSTN⁺ CAF-associated modules forming distinct but interconnected subnetworks (Fig. 3H).

Further examination of the network topology revealed that these POSTN⁺CAF-enriched modules exhibited particularly dense connectivity patterns, indicating strong co-regulatory relationships among their constituent genes. The identification of these core regulatory genes provides valuable insights into the molecular mechanisms that define POSTN⁺CAF identity and function within the tumor microenvironment.

Collectively, these findings define three distinct POSTN⁺CAF-associated gene modules and identify their core regulatory genes, establishing a comprehensive co-expression framework that enhances our understanding of CAF subpopulation specialization in ccRCC. The network-based approach reveals the complex transcriptional architecture underlying CAF heterogeneity and provides a foundation for future investigations into specific pathway activities and regulatory mechanisms.

### Trajectory and transcriptional regulation reveal GATA6-driven differentiation of POSTN⁺ CAFs

**Fig. 4:**
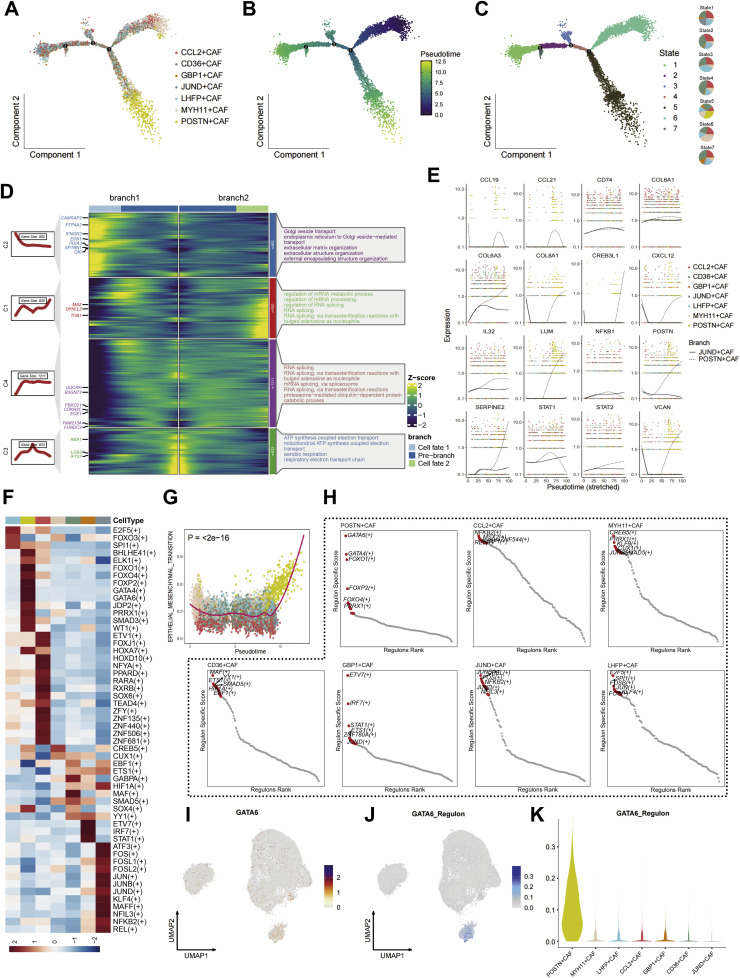
**Trajectory and transcriptional regulatory analysis reveal GATA6-driven differentiation of POSTN⁺ CAFs. (A)** Pseudotime trajectory of CAF subsets reconstructed by *Monocle2*, showing a continuous differentiation process from early-stage CCL2⁺/CD36⁺ CAFs to late-stage POSTN⁺ CAFs. **(B)** Pseudotime color gradient indicating the temporal progression of CAF maturation. **(C)** State classification along the trajectory, defining seven major transcriptional states corresponding to distinct CAF phenotypes. **(D)** Heatmap of branched gene expression dynamics, identifying two major differentiation branches: one enriched for ECM organization and collagen biosynthesis (branch 2) and the other for secretory and metabolic functions (branch 1). Representative GO terms for each branch are shown on the right. **(E)** Expression trends of representative genes across pseudotime, illustrating the sequential upregulation of ECM-associated genes (COL1A1, COL6A3, LUM, POSTN) and transcriptional regulators (STAT1, STAT2, VCAN) during fibroblast activation. **(F)** Heatmap of transcription factor (TF) regulon activities inferred by *pySCENIC*, showing distinct TF usage patterns across CAF subsets. **(G)** Spearman correlation analysis between pseudotime and EMT score, with different colors representing distinct CAF subtypes (p < 2e−16). **(H)** Regulon specificity scores (RSS) identifying top transcription factors enriched in each CAF subtype. GATA6 and FOXF2 were highly specific for POSTN⁺ CAFs, while ETV1 and RFX7 were dominant in GBP1⁺ and JUND⁺ CAFs, respectively. **(I–J)** UMAP visualizations showing GATA6 expression (I) and regulon activity (J) across fibroblast subpopulations, demonstrating specific enrichment in POSTN⁺ CAF clusters. **(K)** Violin plot showing GATA6 regulon activity across CAF subsets, with the highest activity in POSTN⁺ CAFs, supporting its role as a master regulator driving fibroblast differentiation and ECM remodeling.

To elucidate the differentiation hierarchy of fibroblast subsets in ccRCC, we performed pseudotime trajectory reconstruction using the Monocle2 algorithm (Fig. 4A–C). The inferred lineage topology revealed a bifurcating trajectory, in which quiescent LHFP⁺ and CD36⁺ CAFs occupied the root, while JUND⁺ CAFs represented an intermediate state transitioning toward terminal POSTN⁺ CAFs. The pseudotime ordering suggested a progressive activation continuum from resting fibroblasts to ECM-producing and immunomodulatory phenotypes.

Branch-dependent differential expression analysis identified distinct transcriptional programs associated with each fate (Fig. 4D). Branch 1, enriched for JUND⁺ CAFs, displayed upregulation of inflammatory genes such as CCL19, CXCL12, IL32, and SERPINE2, indicating a cytokine- and chemokine-driven iCAF-like state. In contrast, Branch 2, which terminated in POSTN⁺ CAFs, exhibited high expression of COL1A1, COL3A1, LUM, VCAN, and POSTN itself, reflecting a pro-fibrotic myCAF program (Fig. 4E). Pathway enrichment analyses of branch-specific genes highlighted the activation of ECM organization, TGF-β signaling, and oxidative phosphorylation in Branch 2, consistent with metabolic and matrix-remodeling reprogramming (Fig. 4D).

To identify transcriptional regulators governing fibroblast differentiation, we integrated pySCENIC regulon analysis with pseudotime inference. Heatmap profiling revealed dynamic activation patterns of multiple transcription factors during CAF differentiation (Fig. 4F). Spearman correlation analysis demonstrated a strong positive association between pseudotime progression and epithelial-mesenchymal transition (EMT) scores across all CAF subtypes, with particularly pronounced correlations observed in the POSTN⁺ CAF population (p < 2e−16) (Fig. 4G). This robust correlation suggests that EMT-like processes are intrinsically linked to the temporal dynamics of fibroblast maturation in the ccRCC microenvironment. GATA6 emerged as the dominant regulator within POSTN⁺ CAFs, ranking as the top regulon by regulon specificity score (RSS) across all subtypes (Fig. 4H). GATA6 target genes— including POSTN, COL3A1, and ACTA2—were significantly enriched in matrix-related pathways, suggesting that GATA6 orchestrates the transcriptional program driving fibroblast activation and ECM deposition.

Single-cell resolution analysis further elucidated the specific expression patterns of GATA6 across fibroblast subpopulations. UMAP visualization demonstrated that GATA6 mRNA expression was predominantly enriched within POSTN⁺ CAF clusters compared to other fibroblast subsets (Fig. 4I). Consistent with its transcriptional expression, GATA6 regulon activity analysis revealed a similar distribution pattern, with the highest regulon activity scores concentrated specifically in POSTN⁺ CAF populations (Fig. 4J). Quantitative assessment through violin plot analysis confirmed the significantly elevated GATA6 regulon activity in POSTN⁺ CAFs compared to all other fibroblast subtypes (Fig. 4K).These findings collectively delineate a differentiation continuum from quiescent fibroblasts to GATA6-activated POSTN⁺ CAFs, establishing GATA6 as a key transcriptional regulator of stromal remodeling and the formation of the immune-excluded niche in ccRCC.

### POSTN⁺ CAFs shape a hypoxic and immune-excluded microenvironment

**Fig. 5:**
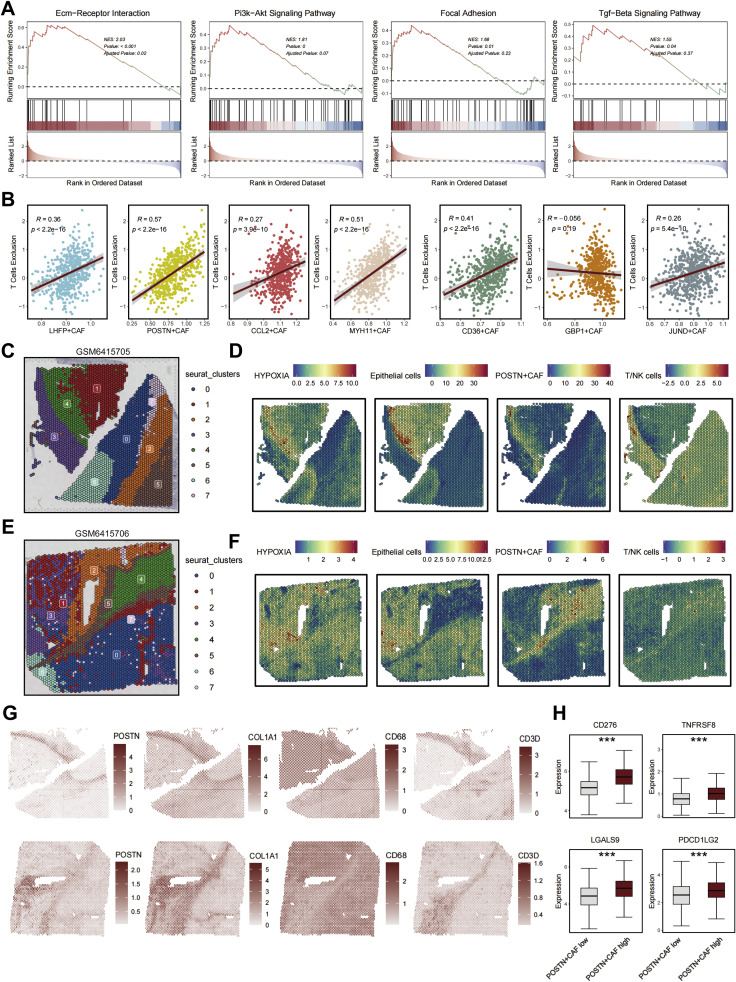
POSTN⁺ CAFs shape an ECM-rich, immune-excluded microenvironment in ccRCC. **(A)** Gene set enrichment analysis (GSEA) of POSTN⁺ CAF–specific genes showing significant activation of ECM–receptor interaction, PI3K–AKT, focal adhesion, and TGF-β signaling pathways, suggesting a pro-fibrotic and matrix-remodeling phenotype. **(B)** Correlation analysis between CAF subtype scores and T-cell exclusion index in the TCGA-KIRC cohort. POSTN⁺, MYH11⁺, and CCL2⁺ CAFs exhibit the strongest positive correlation with T-cell exclusion (Pearson’s *R* = 0.57, 0.51, and 0.49; all *p* < 2.2 × 10⁻¹⁶), supporting their role in forming immune barriers. **(C–F)** Spatial transcriptomic maps from two representative ccRCC samples (GSM6415705 and GSM6415706). Clustering maps (**C, E**) display the spatial organization of tumor and stromal regions, while spatial feature plots (**D, F**) illustrate the co-localization of hypoxia, epithelial regions, POSTN⁺ CAFs, and T/NK cells. POSTN⁺ CAFs accumulate predominantly at the hypoxic tumor–stroma interface, where T/NK-cell infiltration is minimal. **(G)** Spatial expression maps of POSTN, COL1A1, CD68, and CD3D in serial sections, showing co-localization of POSTN and COL1A1 with macrophage-enriched (CD68⁺) but T-cell–depleted (CD3D⁺) regions, consistent with an immune-excluded stromal niche. **(H)** Boxplots comparing the expression of immune checkpoint molecules between POSTN⁺ CAF–high and –low regions. POSTN⁺ CAF–high areas exhibit elevated CD276 (B7-H3), TNFRSF8 (CD30), LGALS9 (Galectin-9), and PDCD1LG2 (PD-L2) levels (**p < 0.0001**), indicating a highly immunosuppressive microenvironment.

To define the functional impact of POSTN⁺ CAFs on the tumor immune landscape, we first conducted gene set enrichment analysis (GSEA) based on bulk transcriptomic data from ccRCC cohorts. POSTN⁺ CAF signature enrichment was positively associated with multiple pro-fibrotic and signaling pathways, including ECM–receptor interaction, PI3K–AKT, focal adhesion, and TGF-β signaling (Fig. 5A). These pathways collectively regulate extracellular matrix organization, cell adhesion, and stromal activation, underscoring the role of POSTN⁺ CAFs as key mediators of tissue remodeling and fibrosis.

We next evaluated the relationship between CAF subtypes and T-cell exclusion scores across bulk datasets. Correlation analyses revealed that POSTN⁺ CAFs exhibited the strongest positive correlation with T-cell exclusion (R = 0.57, p < 2.2 × 10⁻¹⁶), followed by MYH11⁺, CCL2⁺, and CD36⁺ CAFs (Fig. 5B). In contrast, quiescent LHFP⁺ CAFs displayed weak or negative correlations, suggesting that matrix-activated CAF subsets are the dominant contributors to immune exclusion in ccRCC.

To spatially contextualize these findings, we analyzed two representative spatial transcriptomics samples (GSM6415705 and GSM6415706). In both datasets, POSTN⁺ CAFs colocalized with hypoxic regions and epithelial compartments, forming a dense stromal rim that spatially separated T/NK-cell–enriched immune zones from tumor nests (Fig. 5C–F). Hypoxia scoring confirmed that POSTN⁺ CAF-dense regions overlapped with areas of oxygen deprivation, supporting a model in which stromal remodeling and metabolic stress jointly reinforce the immune-excluded phenotype.

Consistent with these spatial features, genes encoding extracellular matrix components (POSTN, COL1A1) and macrophage markers (CD68) were spatially co-expressed within these fibrotic margins, whereas the T-cell marker CD3D was largely restricted to adjacent immune-rich areas (Fig. 5G). This reciprocal spatial segregation highlights the physical barrier function of POSTN⁺ CAFs.

At the molecular level, tumors with high POSTN⁺ CAF signature activation exhibited significantly upregulated immune checkpoint molecules, including CD276 (B7-H3), TNFRSF8 (CD30), LGALS9, and PDCD1LG2 (PD-L2) (Fig. 5H). This pattern suggests that POSTN⁺ CAF accumulation not only establishes a physical barrier to T-cell infiltration but also promotes an immunoregulatory milieu characterized by checkpoint upregulation and T-cell dysfunction.

Collectively, these findings indicate that POSTN⁺ CAFs define a hypoxic, fibrotic, and immune-excluded microenvironment in ccRCC. Through ECM deposition, spatial segregation, and immunoregulatory signaling, these fibroblasts serve as pivotal stromal gatekeepers that restrict immune cell infiltration and foster resistance to immune checkpoint blockade.

### CAF–macrophage interaction network identifies a POSTN⁺ CAF–CCL3⁺ macrophage axis driving immune exclusion

**Fig. 6:**
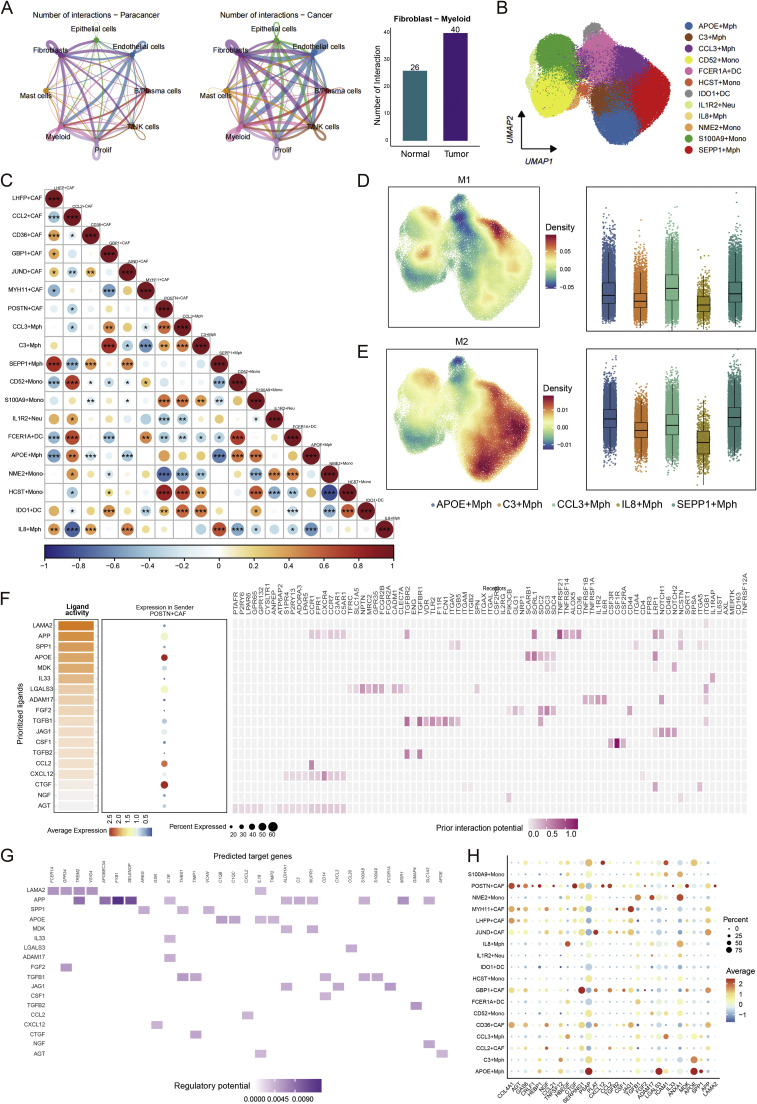
Crosstalk between POSTN⁺ CAFs and CCL3⁺ macrophages defines a pro-fibrotic and immunosuppressive signaling axis. **(A)** Cell–cell communication networks inferred by *CellChat* showing significantly increased fibroblast–myeloid interactions in tumor tissues compared with paracancerous counterparts (40 vs. 26 total interactions). **(B)** UMAP visualization of major myeloid populations identified from single-cell data, including macrophages (CCL3⁺, SEPP1⁺, APOE⁺), monocytes (CD52⁺, NME2⁺), dendritic cells (FCER1A⁺, IL1B⁺, IDO1⁺), and neutrophils (HCST⁺). **(C)** Correlation matrix of CAF and myeloid subtypes based on ligand–receptor signaling intensity. POSTN⁺ CAFs exhibit the strongest interaction with CCL3⁺ macrophages, suggesting preferential CAF–macrophage coupling within the tumor microenvironment.*p < 0.05,**p < 0.01,***p <0.001. **(D–E)** Density distribution and abundance comparison of M1-like and M2-like macrophage subpopulations. M2-like macrophages (CCL3⁺, IL8⁺, SEPP1⁺) are predominant in tumor regions and co-localize with POSTN⁺ CAFs, consistent with an immunosuppressive phenotype. **(F)***NicheNet*-based prediction of ligand–target communication from POSTN⁺ CAFs to macrophages. The top ligands (e.g.,LAMC1, APP, APOE, MDK, SPP1, CCL2, and TGF-β1) display high activity scores and potential to activate macrophage receptors such as TGFBR2, SDC3, and ITGB1. **(G)** Heatmap showing potential target genes in CCL3⁺ macrophages that may be regulated by ligands derived from POSTN⁺ fibroblasts, along with corresponding ligand-receptor interactions. **(H)** Dot plot showing the expression patterns of top 30 ligand genes across all myeloid subtypes and CAF subpopulations, with dot size representing expression percentage and color intensity indicating expression level.

To systematically dissect stromal–immune interactions in ccRCC, we performed cell–cell communication analysis using CellChat and NicheNet frameworks. Global interaction mapping revealed that fibroblast–myeloid communication was markedly increased in tumor tissues compared to paracancerous counterparts (49 vs. 26 significant pairs; Fig. 6A), underscoring the centrality of this stromal–immune axis in the tumor microenvironment.

Reclustering of myeloid populations identified nine distinct subtypes, including APOE⁺ macrophages, C3⁺ macrophages, CCL3⁺ macrophages, IL8⁺ macrophages, SEPP1⁺ macrophages, and several monocyte/DC subsets (Fig. 6B). Correlation matrix analysis demonstrated a strong transcriptional coupling between POSTN⁺ CAFs and CCL3⁺ macrophages, whereas quiescent fibroblast subsets (e.g., LHFP⁺ CAFs) showed weak or inverse correlations (Fig. 6C). The two macrophage modules most closely associated with CAF activation, M1 and M2, were enriched in CCL3⁺ and SEPP1⁺ macrophages (Fig. 6D–E), both characterized by profibrotic and immunosuppressive signatures.

To identify specific molecular mediators of POSTN⁺ CAF-macrophage communication, we employed NicheNet-based ligand-receptor inference. This analysis prioritized several POSTN⁺ CAF-derived ligands with high predicted regulatory potential toward macrophages, including extracellular matrix components (LAMC1, APP, SPP1), signaling molecules (APOE, MDK, TGFB1), and chemotactic factors (CTGF, CXCL12). Dot plot visualization illustrated the expression patterns of these top-ranked ligands across different CAF subtypes, confirming their specific enrichment in POSTN⁺ CAFs. Further examination of ligand-receptor pairs between SPP1⁺ macrophages and POSTN⁺ CAFs revealed organized interaction networks, while heatmap analysis comprehensively validated the strong regulatory potential specifically linking POSTN⁺ CAFs with CCL3⁺ macrophages (Fig. 6F-G).

Expanding our analysis to a broader ligand repertoire, we profiled the expression patterns of the top 30 ligand genes—including extracellular matrix regulators (COL4A1, CTGF, LAMA2), growth factors (HBEGF, FGF2, TGFB1, TGFB2), immunomodulators (CCL21, CCL2, CXCL12, IL33), and diverse signaling molecules (GAS6, NGF, PSAP, APOE, SPP1)—across all myeloid and CAF subpopulations. This comprehensive profiling identified distinct expression hierarchies and suggested multiple candidate pathways that may orchestrate the specialized crosstalk between POSTN⁺ CAFs and their myeloid partners within the ccRCC microenvironment (Fig. 6H).

Collectively, these results uncover a spatially organized POSTN⁺ CAF–CCL3⁺ macrophage axis as the central stromal–immune circuit in ccRCC. Through reciprocal cytokine and ECM signaling, this fibro-myeloid partnership orchestrates stromal remodeling and immune evasion, providing a mechanistic framework for therapeutic strategies targeting both CAF and macrophage compartments.

### Clinical relevance of the POSTN⁺ CAF–CCL3⁺ macrophage axis in prognosis and immunotherapy response

**Fig. 7:**
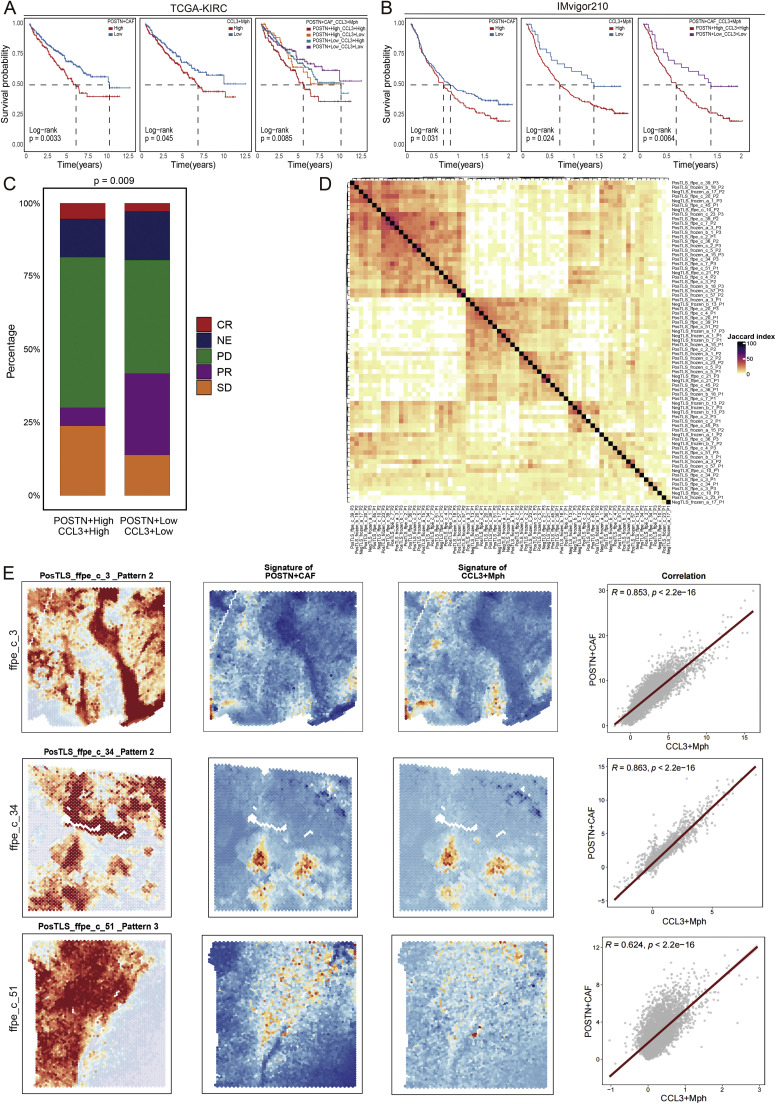
Clinical and spatial validation of the POSTN⁺ CAF–CCL3⁺ macrophage axis as a determinant of **immunotherapy resistance and immune exclusion. (A)** Kaplan–Meier survival analyses in the TCGA-KIRC cohort showing that patients with high POSTN⁺ CAF infiltration exhibit significantly poorer overall survival (*p* = 0.0033). Co-activation of POSTN⁺ CAFs and CCL3⁺ macrophages further worsens prognosis (*p* = 0.0085), indicating synergistic impact on tumor progression. **(B)** Validation in the IMvigor210 cohort (anti–PD-L1–treated urothelial carcinoma) demonstrating that high POSTN⁺ CAF or combined POSTN⁺ CAF/CCL3⁺ macrophage signature correlates with reduced survival and decreased therapeutic response (*p* < 0.05 in all comparisons). **(C)** Immunotherapy response distribution across subgroups stratified by POSTN⁺ CAF and CCL3⁺ macrophage activation. The POSTN⁺High/CCL3⁺High group shows markedly higher rates of progressive disease (PD) and non-response (NE), whereas the POSTN⁺Low/CCL3⁺Low group is enriched for partial (PR) or complete responses (CR) (*p* = 0.009, χ² test). **(D)** Spatial similarity heatmap of transcriptomic patterns in ccRCC tissues, constructed using SpaGene-based Jaccard index analysis, revealing a distinct co-enrichment module corresponding to POSTN⁺ CAF and CCL3⁺ macrophage signatures. **(E)** Spatial transcriptomic validation across multiple tumor regions showing spatial co-localization of POSTN⁺ CAF and CCL3⁺ macrophage signatures at the invasive front and peritumoral stroma. These fibroblast–macrophage niches coincide with tertiary lymphoid structure (TLS)–negative areas, reflecting immune-excluded zones. Scatterplots (right) display strong positive correlations between POSTN⁺ CAF and CCL3⁺ macrophage spatial scores (R = 0.85–0.52, *p* < 2.2 × 10⁻¹⁶), confirming their coordinated organization within the immune-excluded tumor microenvironment.

To evaluate the clinical significance of the POSTN⁺ CAF–CCL3⁺ macrophage axis, we analyzed its association with patient outcomes and immunotherapy response across multiple datasets. In the TCGA–KIRC cohort, patients with high POSTN⁺ CAF signature exhibited significantly poorer overall survival compared to the low group (p = 0.0033; Fig. 7A, left). A similar trend was observed for CCL3⁺ macrophage enrichment (p = 0.045; Fig. 7A, middle). Notably, combined stratification based on both signatures identified a distinct high-risk POSTN⁺/CCL3⁺ dual-high group with the worst prognosis (p = 0.0085; Fig. 7A, right).

These findings were independently validated in the IMvigor210 immunotherapy cohort, where patients with elevated POSTN⁺ CAF and CCL3⁺ macrophage scores also showed shorter overall survival and lower therapeutic benefit under PD-L1 blockade (Fig. 7B). Across both datasets, axis activation was consistently associated with immune-excluded phenotypes and reduced clinical benefit from immune checkpoint inhibitors (ICIs).

Further examination of immunotherapy response distribution revealed that patients in the POSTN⁺High/CCL3⁺High group were markedly enriched in progressive disease (PD) and stable disease (SD) categories, whereas partial (PR) and complete responders (CR) were more frequent in the dual-low group (p = 0.009; Fig. 7C). These data highlight the predictive potential of this stromal–immune signature in identifying ICB-resistant tumors.

To assess the spatial relationship between POSTN⁺ CAFs and CCL3⁺ macrophages, we performed SpaGene-based spatial pattern analysis across 24 tumor sections. Co-expression correlation mapping revealed a high spatial concordance between POSTN⁺ CAF and CCL3⁺ macrophage signatures (R = 0.85–0.52, p < 2.2 × 10⁻¹⁶; Fig. 7E), suggesting that these two cell types preferentially co-localize within the immune-excluded tumor margins. The Jaccard index heatmap further demonstrated consistent clustering of ECM- and myeloid-dominant spatial modules (Fig. 7D), reinforcing the existence of a conserved fibro–myeloid microenvironmental architecture across ccRCC samples.

Spatial visualization of representative sections confirmed that POSTN⁺ CAF and CCL3⁺ macrophage signals co-accumulate at the invasive front, forming a dense stromal boundary juxtaposed to tertiary lymphoid structures (TLSs) (Fig. 7E). This pattern delineates a spatial dichotomy between immune-rich (TLS-associated) and immune-cold (CAF/macrophage-dense) regions, indicating that the fibro-myeloid axis contributes to immune segregation within the tumor microenvironment.

Collectively, these clinical and spatial data establish the POSTN⁺ CAF-CCL3⁺ macrophage axis as a prognostic and predictive biomarker in ccRCC. Its activation is linked to poor survival, reduced responsiveness to immune checkpoint therapy, and the formation of spatially organized immune-excluded niches. These findings suggest that disrupting this fibro–myeloid signaling circuit could serve as a therapeutic strategy to restore immune infiltration and improve immunotherapy efficacy in ccRCC.

## Discussion

This study reveals a spatially organized stromal-immune mechanism underlying immune exclusion in ccRCC. Through integrative single-cell and spatial transcriptomic analyses, we identified a distinct POSTN⁺ CAF-CCL3⁺ macrophages axis that defines the fibrotic and immune-excluded tumor microenvironment by colocalizing at the tumor-stroma interface and cooperatively remodeling the ECM to restrict CD8⁺ T-cell infiltration. Mechanistically, hypoxia and TGF-β/STAT3 signaling drive CAFs to mature into ECM-dominant POSTN⁺ phenotypes. These fibroblasts secrete periostin to recruit and polarize CCL3⁺ macrophages, which release TGF-β, PDGFB, and SPP1 to sustain fibrosis and immune exclusion. Spatial transcriptomic confirmed the colocalization of POSTN⁺ CAF-CCL3⁺ macrophages niche at immune-excluded margins, forming a stromal boundary that opposes TLSs and spatially separates immune-rich and immune-cold zones within tumors. Clinically, activation of this axis correlates with resistance to ICB, and poor prognosis in ccRCC. Collectively, our findings uncover a key stromal-immune circuit driving immune exclusion and provide a mechanistic foundation for enhancing immunotherapy efficacy.

Advances in single-cell transcriptomics have enabled precise characterization of CAF heterogeneity in the tumor microenvironment[25,26]. Based on canonical markers, our study identified seven CAF subsets in ccRCC: CCL2⁺, CD36⁺, GBP1⁺, JUND⁺, LHFP⁺, MYH11⁺, and POSTN⁺ CAFs. Among these, POSTN⁺ CAFs were selectively enriched in tumor tissues and exhibited strong activation of ECM-receptor interaction, focal adhesion, TGF-β, and PI3K-AKT pathways, indicating a matrix-remodeling and pro-fibrotic phenotype[27,28]. Within the established CAF taxonomy, POSTN⁺ CAFs most closely align with the myCAF phenotype, as supported by their enrichment of myCAF-associated transcriptional signatures and high expression of canonical contractile and extracellular matrix–related markers. Notably, compared with previously described myCAF populations, POSTN⁺ CAFs exhibit a more pronounced hypoxia-adapted and STAT3-associated transcriptional program and preferentially localize to the tumor-stroma interface, suggesting that they represent a spatially specialized myCAF subset optimized for stromal remodeling and immune exclusion in ccRCC. Their spatial localization at the tumor-stroma interface further suggested a central role in shaping the immune-excluded microenvironment[29]. POSTN encodes periostin, a secreted matricellular glycoprotein that modulates cell adhesion, migration, and ECM organization during tissue remodeling and tumor progression[30,31]. Through the secretion of periostin, POSTN⁺ CAFs interact with integrin receptors αvβ3 and αvβ5, triggering FAK-PI3K-AKT and STAT3 signaling cascades that promote fibroblast activation, collagen network organization, and the formation of a mechanically reinforced, immunosuppressive stromal niche[32,33]. These features indicate that POSTN⁺ CAFs act as key structural organizers linking fibrosis with immune modulation, establishing the stromal context in which macrophage activation and T-cell exclusion occur.

Within this stromal framework, macrophages emerge as the dominant immune counterparts sustaining fibroblast-driven immune suppression[34]. Among multiple myeloid populations, CCL3⁺ macrophages showed the strongest transcriptional and interaction correlation with POSTN⁺ CAFs in ccRCC, supporting a preferential coupling between these two compartments. CCL3, a chemokine known to signal via CCR1 and CCR5, has been implicated in myeloid recruitment, macrophage polarization, and local immune modulation[35,36]. Meanwhile, CCL3⁺ macrophages in our data were enriched for genes associated with inflammatory chemokines, angiogenesis, and immunosuppression, including SPP1, VEGFA, IL10, and TGFB1[37]. Ligand-receptor and regulatory network inference further suggested that POSTN⁺ CAF-derived signals, such as IL-6, SPP1 and CCL2, converge on STAT3- and CREB3L1-driven programs in CCL3⁺ macrophages, stabilizing this protumor phenotype[38]. In turn, CCL3⁺ Macrophages secrete TGF-β, PDGFB and SPP1, providing reciprocal cues that enhance fibroblast activation and extracellular matrix production. Together, these data support a model in which CCL3⁺ Macrophages function as the immunoregulatory arm of the POSTN⁺ CAF niche, locking in a fibrotic, macrophage-dominated microenvironment that is refractory to effective T-cell mediated immunity. Although CCL3 serves as a defining marker of this macrophage subset, its functional role in the POSTN⁺ CAF-associated niche is likely dominated by paracrine signaling. Ligand-receptor inference suggests that CAF-derived cues may facilitate the recruitment and spatial retention of CCL3⁺ macrophages, thereby reinforcing the fibro-myeloid niche. An autocrine role of CCL3 in sustaining macrophage activation cannot be excluded and may cooperate with CAF-derived cytokines to stabilize this immunosuppressive microenvironment.

Building on these findings, therapeutic strategies targeting the CAF-macrophage symbiotic loop have demonstrated promising potential across multiple tumor types. In hepatocellular carcinoma, POSTN⁺ CAFs interact with SPP1⁺ macrophages through the IL-6/STAT3 axis, forming a fibro-myeloid feedback loop that reinforces fibrosis and limits CD8⁺ T-cell infiltration. Inhibition of this signaling by POSTN knockdown or IL-6/STAT3 blockade restores immune accessibility and enhances the efficacy of anti-PD-1 therapy[39]. These findings provide a mechanistic basis for targeting both stromal and myeloid compartments to overcome immune exclusion. Although POSTN itself is a secreted matricellular protein and may be challenging to target directly, our NicheNet analysis highlights downstream mediators such as IL-6, TGF-β, and SPP1 as more tractable intervention nodes within the POSTN⁺ CAF-CCL3⁺ macrophage axis. Therapeutic prioritization among these pathways is likely context dependent and must balance efficacy with potential toxicity, given the physiological roles of fibroblasts and macrophages in tissue repair and immune homeostasis. Notably, similar fibro-myeloid programs and ECM-driven immune exclusion have been reported in other tumor types, suggesting that this axis may represent a broader stromal mechanism rather than a ccRCC-exclusive feature. CAF-directed therapies aim to disrupt the fibrotic barrier and relieve stromal rigidity: combining IL-6 blockade with PD-1/PD-L1 inhibitors enhances T-cell infiltration[40,41], whereas FAK or TGF-β inhibition mitigates matrix deposition and re-sensitizes tumors to immunotherapy[[42], [43], [44]]. Macrophage-targeting strategies instead focus on reprogramming rather than depletion. CSF1R inhibitors shift macrophages toward pro-inflammatory phenotypes[45], while CD40 or TLR7/8 agonists activate antigen presentation and synergize with PD-1 blockade to restore antitumor immunity[[46], [47], [48]]. Collectively, these dual-target approaches offer a coherent therapeutic rationale that breaks the stromal barrier and restores immune accessibility by simultaneously dismantling fibrosis and reversing immune suppression. This framework supports the POSTN⁺ CAF-CCL3⁺ macrophage axis as a potential actionable target for stroma-centric immunotherapy in ccRCC.

The novelty of this study lies in integrating single-cell and spatial transcriptomics to resolve stromal-immune interactions in ccRCC. For the first time, we identify a spatially organized POSTN⁺ CAF-CCL3⁺ macrophage axis that drives T-cell exclusion and immunotherapy resistance. Through SpaGene-based pattern analysis across 24 TLS⁺/TLS⁻ samples, we further uncover distinct ECM and immune-dominant spatial programs, linking POSTN⁺ CAF enrichment to the peripheral positioning of TLSs and revealing the structural basis of immune segregation in ccRCC. However, as our conclusions are based on transcription data rather than direct functional evidence, future work should employ *in vitro* and *in vivo* experiments to confirm its mechanistic role in driving immune exclusion. For example, CAF–macrophage co-culture assays could be used to directly assess reciprocal activation and extracellular matrix remodeling, whereas targeted genetic or pharmacologic perturbation of key signaling pathways may help delineate the functional consequences of this axis on immune exclusion. Another limitation of this study is the lack of validation in ccRCC-specific immunotherapy cohorts with available transcriptomic data. Although the IMvigor210 cohort was analyzed as supportive evidence, direct validation of the proposed axis in ccRCC patients treated with immune checkpoint blockade remains constrained by data availability and warrants future investigation. Looking forward, large-scale clinical cohorts of ccRCC patients should evaluate the prognostic and predictive value of the POSTN⁺ CAF-CCL3⁺ macrophage signature, paving the way for biomarker-driven patient stratification. Translating this axis into a therapeutic paradigm by combining stroma-targeted agents that disrupt fibroblast-macrophage signaling with immune checkpoint blockade may ultimately enhance immunotherapy efficacy and enable more personalized treatment for ccRCC.

## Funding statement

N/A

## Data availability statement

The open-access datasets are available on the following URLs: single-cell RNA-seq data (GSE242299, GSE224630, GSE222703, GSE210042, GSE207493, GSE159115, GSE156632) from the GEO database (https://www.ncbi.nlm.nih.gov/geo), the Aleksobrad dataset from GitHub (https://github.com/Aleksobrad/single-cell-rcc-pipeline), bulk transcriptome data (TCGA-KIRC) from the the Cancer Genome Atlas (TCGA) database (https://xena.ucsc.edu), and spatial transcriptomics data (GSE210041, GSE175540) from the GEO database (https://www.ncbi.nlm.nih.gov/geo). Additional data are available upon reasonable request from the corresponding author.

## Ethics declarations

N/A

## CRediT authorship contribution statement

**Yingjian Wang:** Writing – original draft, Visualization, Validation, Software, Resources, Project administration, Methodology, Investigation, Formal analysis, Data curation, Conceptualization. **Bingtong Yue:** Supervision, Software, Resources, Project administration, Methodology, Investigation, Formal analysis, Data curation, Conceptualization. **Hongqiang Ni:** Software, Resources, Project administration, Methodology, Investigation, Formal analysis, Data curation, Conceptualization. **Jinchun Chen:** Validation, Supervision, Software, Resources, Project administration, Methodology, Investigation, Funding acquisition, Formal analysis, Data curation, Conceptualization. **Run Shi:** Validation, Supervision, Software, Resources, Project administration, Methodology, Investigation, Formal analysis, Data curation, Conceptualization. **Zhe Wang:** Supervision, Software, Resources, Project administration, Methodology, Investigation, Formal analysis, Data curation, Conceptualization. **Xinglai Dai:** Visualization, Validation, Supervision, Software, Resources, Project administration, Methodology, Investigation, Formal analysis, Data curation, Conceptualization. **Maolin Sheng:** Writing – review & editing, Validation, Supervision, Resources, Project administration.

## Declaration of competing interest

The authors declare that they have no known competing financial interests or personal relationships that could have appeared to influence the work reported in this paper.

## Appendix Supplementary materials

Image, application 1

Image, application 2

Image, application 3

Image, application 4

Image, application 5

Image, application 6
