# Supplementary material for: POSTN⁺ cancer-associated fibroblast–CCL3⁺ macrophage crosstalk defines the immune-excluded tumor microenvironment in clear cell renal cell carcinoma
Source: Transl Oncol. 2026 Jan 24;65:102682. doi: 10.1016/j.tranon.2026.102682 (PMC12860634; doi:10.1016/j.tranon.2026.102682)
Supplement: Supplementary file 1 [file mmc1.docx]

**Supplementary Table 1. Six CAF subtypes signatures and M1/M2 signatures.**

| **iCAF** | LMNA, GSN, DPT, EFEMP1, IL6, C3, COL14A1, CXCL12, TNXB, OGN, PDPN, HAS1, PDGFRA |
| --- | --- |
| **myCAF** | TAGLN, ACTA2, BGN, TPM2, TPM1, MYL9, CTHRC1, POSTN, INHBA, THBS2, MMP11, FAP |
| **apCAF** | CD74, HLA-DRB1, CXCL12, CCL21, HLA-DRA |
| **pCAF** | AURKA, BUB1, CCNB1, CCND1, CCNE1, DEK, E2F1, FEN1, FOXM1, H2AFZ, HMGB2, MCM2, MCM3, MCM4, MCM5, MCM6, MKI67, MYBL2, PCNA, PLK1, TOP2A, TYMS, ZWINT |
| **vCAF** | MYH11, DSTN, MUSTN1, ADIRF |
| **lpCAF** | APOC3, APOC1, APOA2, APOC2, FABP1 |
| **M1 Macrophage Polarization** | IL12, IL23, TNF, IL6, CD86, MHCII, IL1B, MARCO, iNOS, CD64, CD80, CXCR10, CXCL9, CXCL10, CXCL11, IL1A, TNFa, CCL5, IRF5, IRF1, CD40, IDO1, KYNU, CCR7, CD45, CD68, CD115, HLA-DR, CD205, CD14 |
| **M2 Macrophage Polarization** | ARG1, ARG2, IL10, CD32, CD163, CD23, CD200R1, PD-L2, PDL1, MARCO, CSF1R, CD206, IL1RN, IL1R2, IL4R, CCL4, CCL13, CCL20, CCL17, CCL18, CCL22, CCL24, LYVE1, VEGFA, VEGFB, VEGFC, VEGFD, EGF, CTSA, CTSB, CSTC, CTSD, TGFB1, TGFB2, TGFB3, MMP14, MMP19, MMP9, CLEC7A, WNT7B, FASL, TNFSF12, TNFSF8, CD276, VTCN1, MSR1, FN1, IRF4, CD45, CD68, CD115, HLA-DR, CD205, CD14 |
