# Supplementary figures and images for: POSTN⁺ cancer-associated fibroblast–CCL3⁺ macrophage crosstalk defines the immune-excluded tumor microenvironment in clear cell renal cell carcinoma
Source: Transl Oncol. 2026 Jan 24;65:102682. doi: 10.1016/j.tranon.2026.102682 (PMC12860634; doi:10.1016/j.tranon.2026.102682)

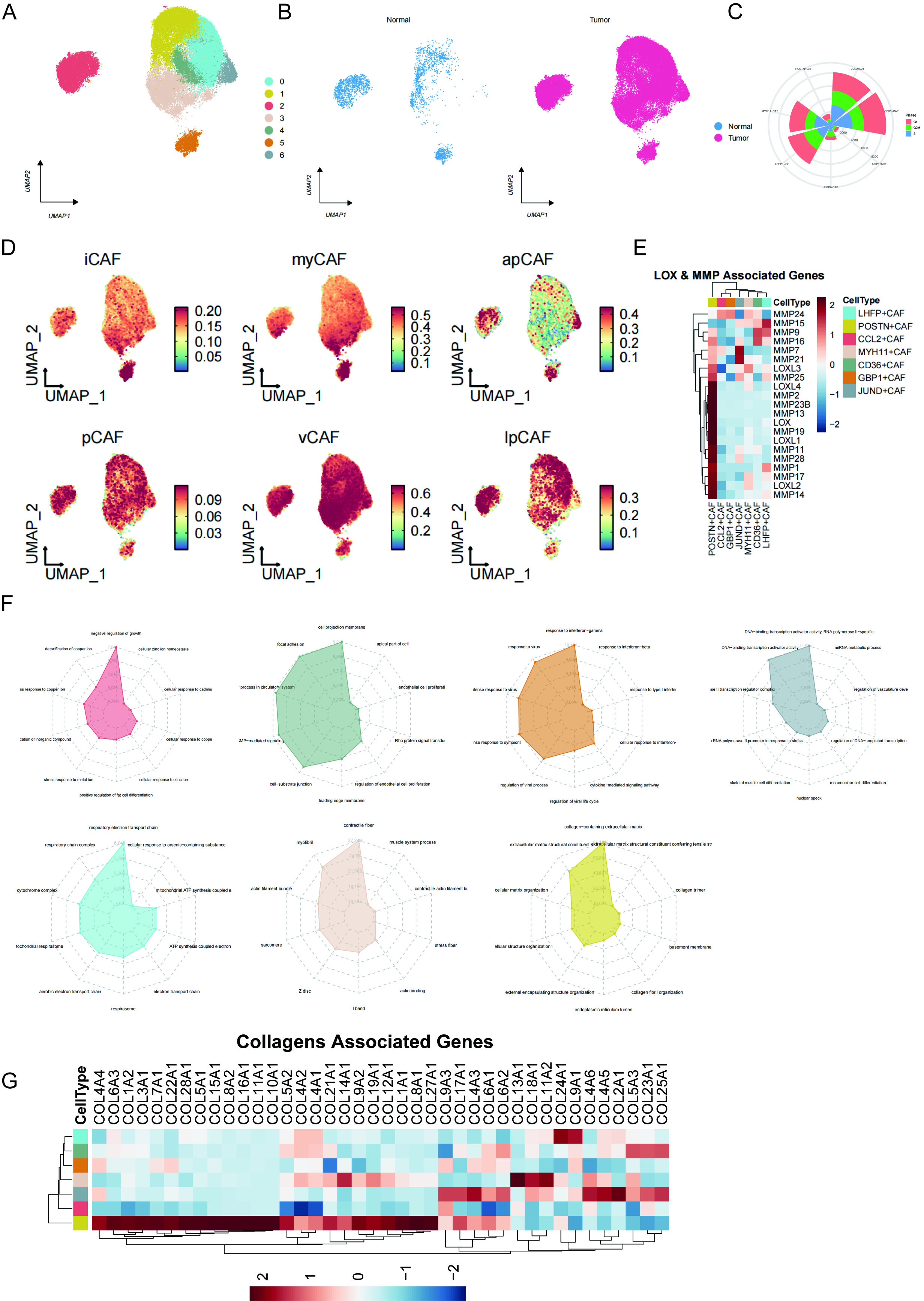

Supplement: Supplementary file 2 [file mmc2.zip › S1.tif]

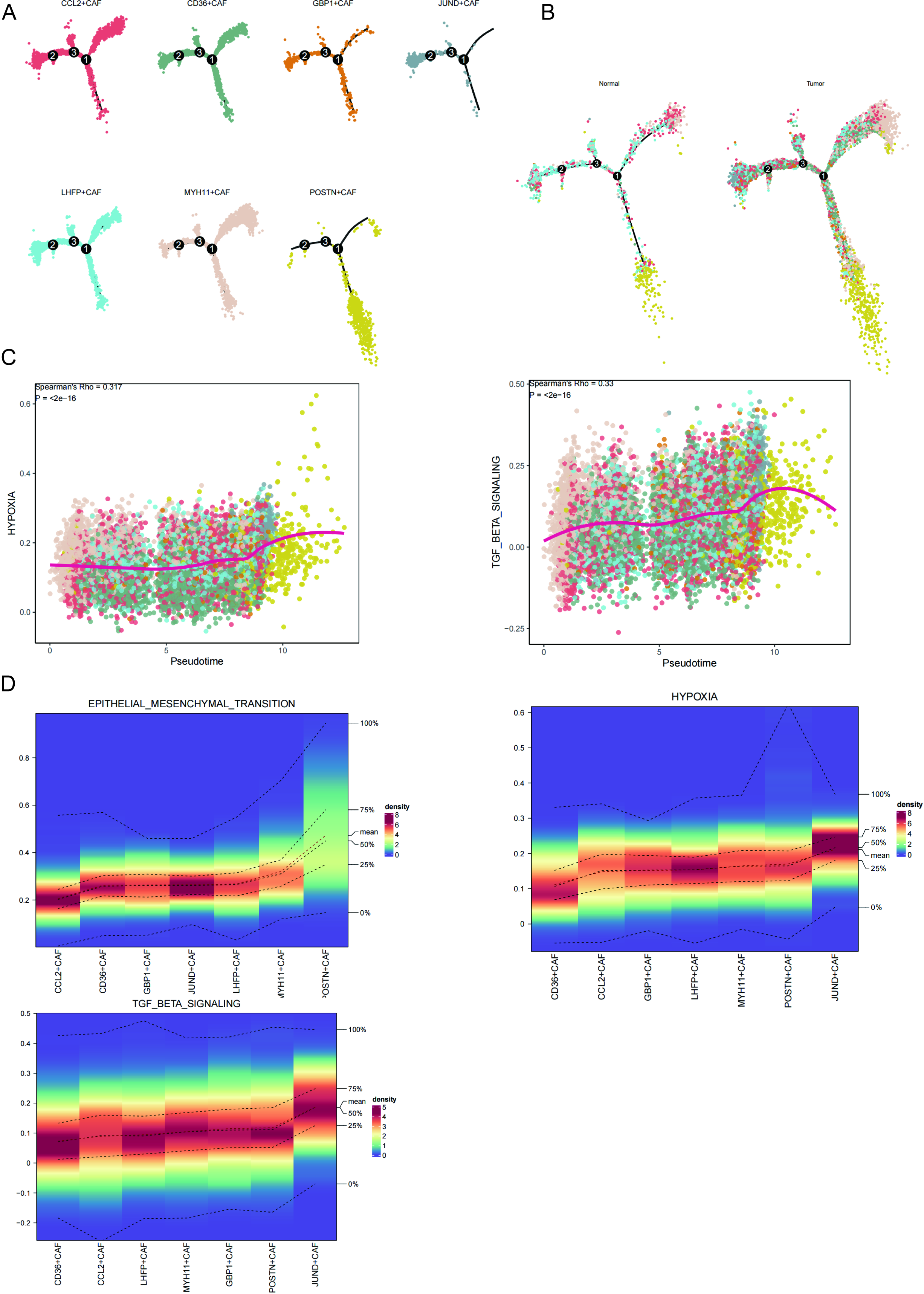

Supplement: Supplementary file 3 [file mmc3.zip › S2.tif]

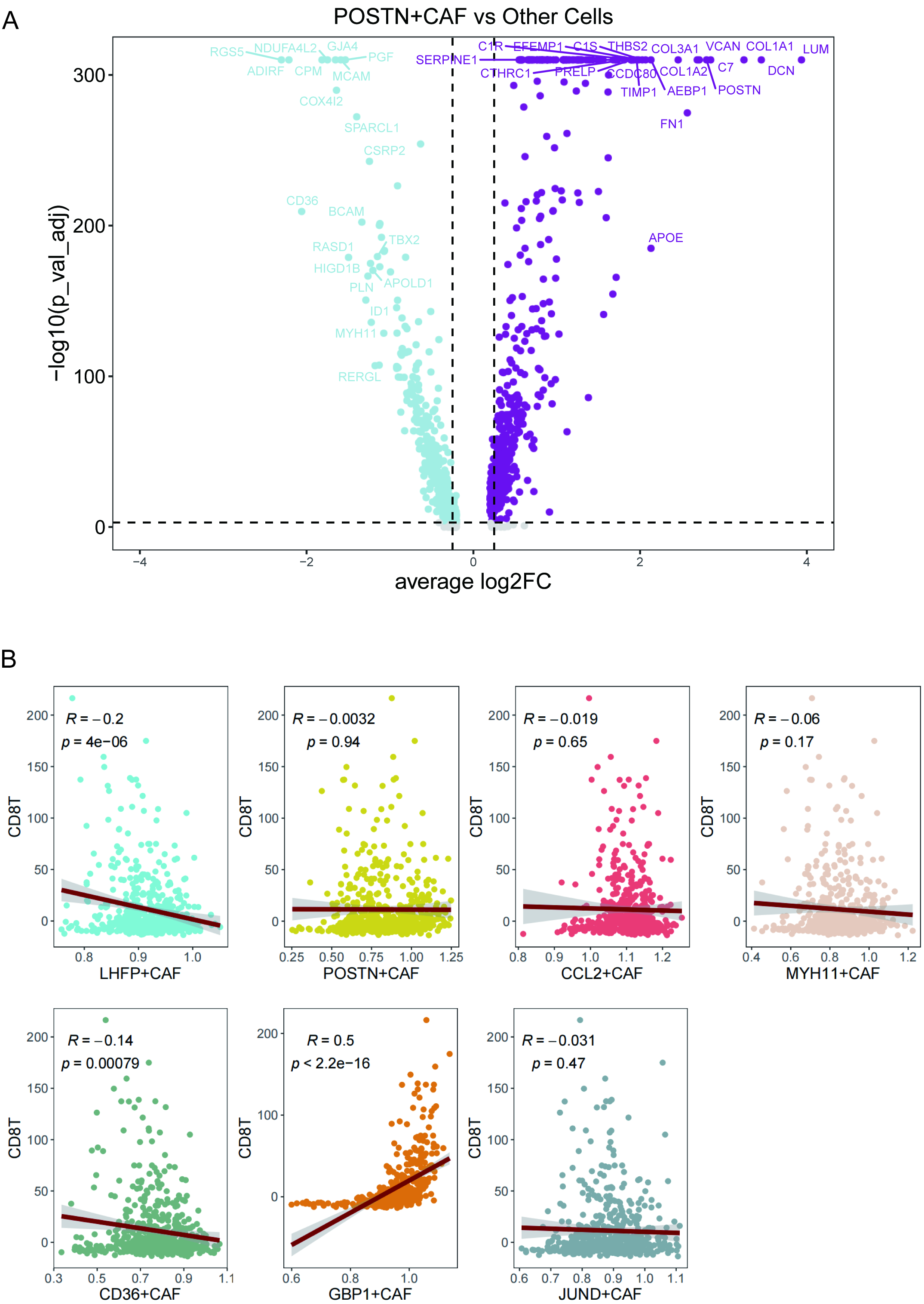

Supplement: Supplementary file 4 [file mmc4.zip › S3.tif]

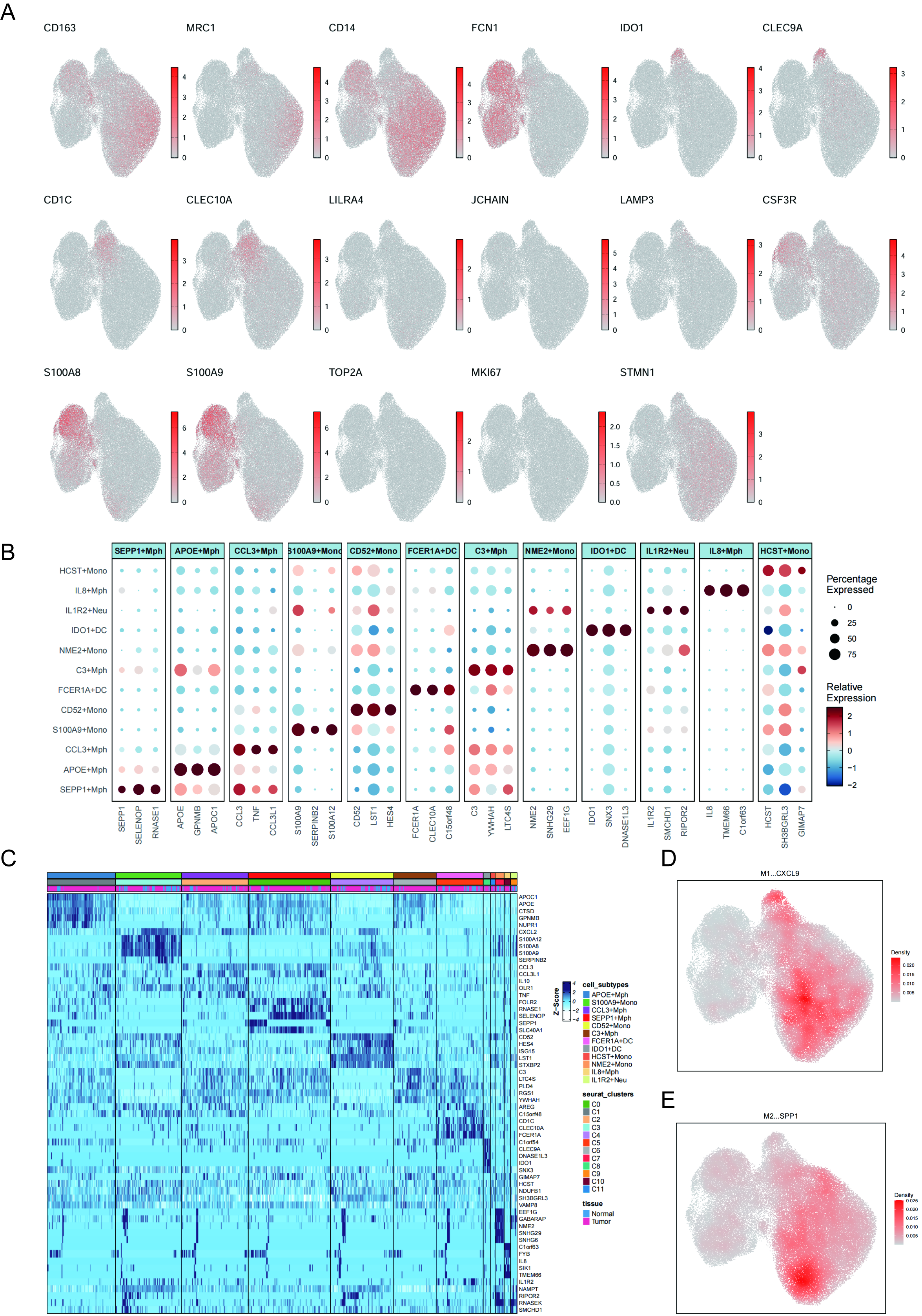

Supplement: Supplementary file 5 [file mmc5.zip › S4.tif]

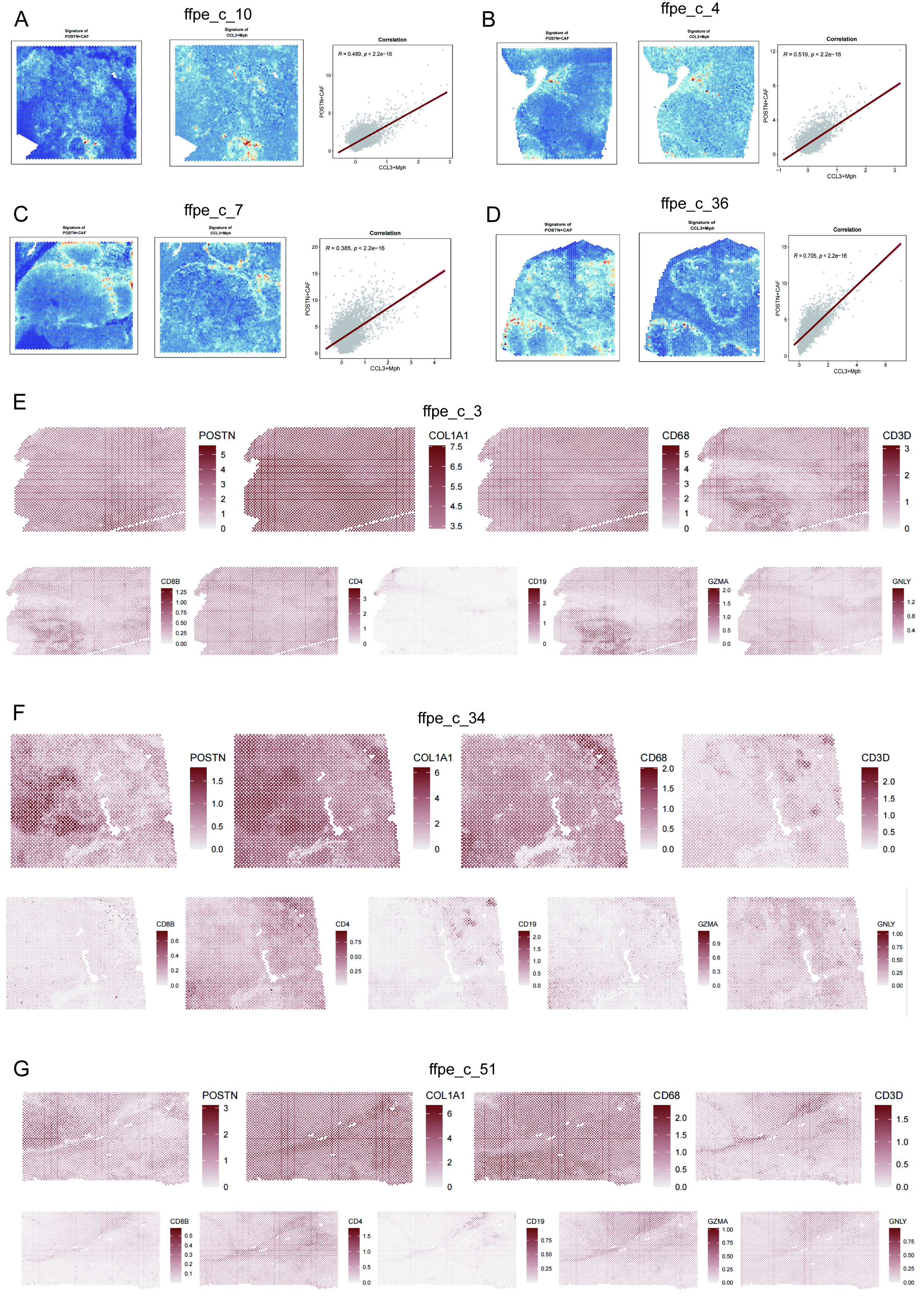

Supplement: Supplementary file 6 [file mmc6.zip › S5.tif]
